# Repetitive Erythropoietin Treatment Improves Long-Term Neurocognitive Outcome by Attenuating Hyperoxia-Induced Hypomyelination in the Developing Brain

**DOI:** 10.3389/fneur.2020.00804

**Published:** 2020-08-12

**Authors:** Monia Vanessa Dewan, Meray Serdar, Yohan van de Looij, Mirjam Kowallick, Martin Hadamitzky, Stefanie Endesfelder, Joachim Fandrey, Stéphane V. Sizonenko, Josephine Herz, Ursula Felderhoff-Müser, Ivo Bendix

**Affiliations:** ^1^Department of Paediatrics I, Neonatology and Experimental Perinatal Neurosciences, University Hospital Essen, University Duisburg-Essen, Essen, Germany; ^2^Division of Child Development and Growth, Department of Paediatrics, School of Medicine, University of Geneva, Geneva, Switzerland; ^3^Center for Biomedical Imaging, Animal Imaging and Technology, Ecole Polytechnique Fédérale de Lausanne, Lausanne, Switzerland; ^4^Institute of Medical Psychology and Behavioural Immunobiology, University Hospital Essen, University Duisburg-Essen, Essen, Germany; ^5^Department of Neonatology, Charité-Universitätsmedizin Berlin, Berlin, Germany; ^6^Institute of Physiology, University Hospital Essen, University Duisburg-Essen, Essen, Germany

**Keywords:** erythropoietin, neuroprotection, preterm brain injury, white matter injury, hyperoxia, myelination

## Abstract

**Introduction:** Preterm infants born before 28 weeks of gestation are at high risk of neurodevelopmental impairment in later life. Cerebral white and gray matter injury is associated with adverse outcomes. High oxygen levels, often unavoidable in neonatal intensive care, have been identified as one of the main contributing factors to preterm brain injury. Thus, preventive and therapeutic strategies against hyperoxia-induced brain injury are needed. Erythropoietin (Epo) is a promising and also neuroprotective candidate due to its clinical use in infants as erythropoiesis-stimulating agent.

**Objective:** The objective of this study was to investigate the effects of repetitive Epo treatment on the cerebral white matter and long-term motor-cognitive outcome in a neonatal rodent model of hyperoxia-induced brain injury.

**Methods:** Three-day old Wistar rats were exposed to hyperoxia (48 h, 80% oxygen). Four doses of Epo (5,000 IU/kg body weight per day) were applied intraperitoneally from P3-P6 with the first dose at the onset of hyperoxia. Oligodendrocyte maturation and myelination were evaluated via immunohistochemistry and Western blot on P11. Motor-cognitive deficits were assessed in a battery of complex behavior tests (Open Field, Novel Object Recognition, Barnes maze) in adolescent and fully adult animals. Following behavior tests animals underwent post-mortem diffusion tensor imaging to investigate long-lasting microstructural alterations of the white matter.

**Results:** Repetitive treatment with Epo significantly improved myelination deficits following neonatal hyperoxia at P11. Behavioral testing revealed attenuated hyperoxia-induced cognitive deficits in Epo-treated adolescent and adult rats.

**Conclusion:** A multiple Epo dosage regimen protects the developing brain against hyperoxia-induced brain injury by improving myelination and long-term cognitive outcome. Though current clinical studies on short-term outcome of Epo-treated prematurely born children contradict our findings, long-term effects up to adulthood are still lacking. Our data support the essential need for long-term follow-up of preterm infants in current clinical trials.

## Introduction

Extremely preterm born infants (<28 weeks of gestation) are at high risk for neurodevelopmental impairment. Around 50% of survivors suffer from neurological or developmental disabilities ranging from various motor, cognitive and sensory impairments to behavioral and emotional disorders in later life (1–3). Cerebral magnetic resonance imaging (MRI) studies at term equivalent age revealed that most survivors show cerebral gray and white matter abnormalities, which are associated with adverse outcome (4). The high risk of neurodevelopmental disabilities is caused by the high vulnerability of the immature brain to the extra-uterine environment in a period of enormous brain growth and maturation (5).

Clinical and pre-clinical studies suggested that the developing brain is particularly vulnerable to chronic exposure to high oxygen levels resulting in subtle and diffuse brain injury (6, 7). Rodent models of hyperoxia-induced brain injury demonstrated that supraphysiologic oxygen-levels induce oxidative stress, inflammation and cellular degeneration, leading to hypomyelination and long-term cognitive deficits (7–10). Despite these findings, oxygen remains an indispensable therapeutic agent in neonatal intensive care. Therefore, neuroprotective agents preventing oxygen-induced brain injury are urgently needed.

One of these promising neuroprotective candidates is Erythropoietin (Epo) (11), an endogenous hormone, which in its recombinant form has been used for the prevention of anemia of prematurity for almost three decades (12). Besides its hematopoietic effects, Epo is suggested to be a neurotrophic and neuroprotective factor in the developing brain, where it is expressed by different cell types such as neurons, oligodendrocytes and astrocytes from early gestation on (13–15). Several studies in experimental models of neonatal brain injury have confirmed its neuroprotective effects (16–19). However, findings from clinical studies are inconsistent: While some retro- and prospective studies revealed improved neurocognitive outcome and white matter development in MRI studies (20–22), recent results from two large randomized controlled trials could not confirm these findings at the corrected age of 2 years (23, 24).

In the rodent model of hyperoxia-induced brain injury, we have previously shown that a single bolus application of 20,000 IE/kg body weight of Epo at the onset of hyperoxia leads to long-lasting improvement of neurocognitive development up to adolescent and adult age (25). Suggested mechanisms include a reduction of oxidative stress, cellular degeneration and inflammation, as well as modulation of autophagy signaling and improvement of synaptic plasticity (16, 25–27). Although bolus Epo-application resulted in a reduced degeneration of oligodendrocytes in our previous study, myelination or white matter development were not changed (25). These results were in contrast to findings in other experimental settings and in clinical studies using multiple dosage regimes (21, 28–30).

Taking into account that in clinical settings, a single-dose Epo regimen is uncommon and that safety-studies have already proven good tolerability of even larger doses (31), the aim of this study was to investigate effects of a repetitive Epo treatment in a model of hyperoxia-induced perinatal brain injury. We hypothesized that repetitive Epo applications protect the developing brain against hyperoxia-induced hypomyelination as well as microstructural alterations of the white matter and ameliorate long-term neurodevelopmental deficits.

## Materials and Methods

### Animals and Experimental Design

All animal procedures were approved by the local animal welfare committee and performed according to the guidelines of the University Hospital Essen. Three-day old (P3) Wistar rat pups were exposed to 80% oxygen for 48 h in an oxygen chamber (OxyCycler, BioSpherix, Lacona, NY, USA). The control group was kept under normoxic conditions (21% oxygen, room-air). In both groups, pups were with their lactating dams. After the first 24 h, dams were exchanged in order to avoid prolonged exposition to hyperoxia. From P3 to P6 animals received daily intraperitoneal (i.p.) injections of 5,000 IU/kg body weight Epo (Epo, NeoRecormon®, Boehringer-La Roche, Grenzach, Germany) culminating in a total dose of 20,000 IU/kg body weight. The first Epo dose was administered at the onset of hyperoxia. Control animals received an equal amount of saline (10 mL/kg body weight).

In total, 132 pups derived from 14 litters were enrolled in this study. Pups per litter were randomly assigned to all treatment groups considering sex–and weight. Increase of bodyweight was monitored daily until P11 and once a week afterwards. No differences were observed between the study groups throughout the entire experiments. Behavioral studies were performed in adolescent (1–2 months old) and adult (4 months old) rats. Pups were sacrificed on P11 (88 pups, 10 litters) and at the age of 5 months (44 pups, 4 litters). Animals sacrificed on P11 were evaluated for subacute white matter injury via Western blot analysis and immunohistochemistry. Pups sacrificed at fully adult age (5 months), which were subjected to behavioral assessment, were evaluated for microstructural changes via diffusion tensor imaging (DTI).

For Western blot analysis, animals were decapitated following transcardial perfusion with phosphate buffered saline. Hemispheres (excluding cerebellum) were snap frozen in liquid nitrogen and stored at 80°C until further processing. For histological and DTI studies, pups were transcardially perfused with phosphate buffered saline followed by 4% paraformaldehyde (PFA, Sigma-Aldrich, Munich, Germany). Brains were post-fixed in 4% PFA overnight at 4°C and embedded in paraffin or sent for DTI.

### Immunoblotting

Snap-frozen brain tissues were homogenized in ice-cooled radioimmunoprecipitation assay (RIPA, Sigma-Aldrich) buffer, phenylmethanesulfonyl fluoride (PMFS, Sigma-Aldrich), complete Mini, EDTA-free (Roche, Basel, Switzerland) and sodium orthovanadate. Homogenates were centrifuged at 17,000 g for 20 min at 4°C. Protein concentrations of the cytosolic fraction were determined using the BCA assay kit (Thermo Fisher Scientific, Dreieich, Germany). Western blotting was performed with 30 μg lysate per sample. Proteins were separated in 15% sodium dodecyl sulfate polyacrylamide gels and transferred to 0.2 μm pore nitrocellulose membranes (Sigma-Aldrich). To control for equal protein transfer, membranes were stained with Ponceau S solution (Sigma Aldrich). Blocking of non-specific protein binding in 5% non-fat dry milk in Tris buffered saline/0.1% Tween 20 (TBS-T) for 60 min at room temperature was followed by primary antibody incubation in 2.5 or 5% non-fat dry milk in TBST at 4°C overnight. The following primary antibodies were used: monoclonal mouse anti-myelin basic protein (MBP) (1:1000, Covance, Münster, Germany), monoclonal mouse anti-oligodendrocyte transcription factor 2 (Olig2) (1:1000, Merck Millipore, Darmstadt, Germany), monoclonal mouse anti-2', 3'-cyclic nucleotide 3'-phosphodiesterase (CNPase) (1:1000, Merck Millipore) and monoclonal rabbit anti-Glycerinaldehyd-3-phosphat-Dehydrogenase (GAPDH) (1:5000, Cell Signaling, Frankfurt, Germany). To detect primary antibody binding, membranes were incubated for 1 h at room temperature with horseradish peroxidase-conjugated secondary anti-mouse (1:5000, Dako, Hamburg, Germany) or anti-rabbit (1:2000, Dako) antibody. Antibody binding was detected by using enhanced chemiluminescence (GE Healthcare Life Sciences, Munich, Germany) and visualized by the ChemiDoc XRS+ imaging system. ImageLab software (Bio-Rad, Munich, Germany) was used for densitometric analysis. Density ratios between the protein of interest and the reference protein GAPDH were calculated for each sample. These ratios were normalized to the control group NO.

### Immunohistochemistry

Ten micrometre coronal sections (−3.72 ± 0.7 mm from bregma) were deparaffinized and rehydrated followed by antigen-retrieval in preheated 10 mM sodium-citrate buffer (pH 6.0) for 30 min. After blocking with 1% bovine serum albumin and 0.3% cold fish skin gelatine in TBST (Sigma-Aldrich) slides were incubated with primary antibodies at 4°C overnight. The following primary antibodies were used: polyclonal rabbit anti-Olig2 (1:100, Millipore), monoclonal rat anti-MBP (1:200, abcam, Berlin, Germany) and monoclonal mouse anti-adenomatous polyposis coli clone CC1 (APC-CC1, 1:100, Merck Millipore). This was followed by incubation with appropriate secondary antibodies for 1 h at room temperature [anti-mouse Alexa Fluor 488, anti-rat Alexa Fluor 555 and anti-rabbit Alexa Fluor 647 (all 1:500, Invitrogen, Darmstadt, Germany)]. Sections were counterstained with 4,6-diamidino-2-phenylindole (DAPI) (1 μg/mL, Invitrogen, Karlsruhe, Germany).

For image acquisition four laser lines (laser diode, 405 nm; Ar laser, 514 nm; G-HeNe laser, 543 nm; Rn Laser 639 nm) and four different filters (450/50-405 LP, 515/20-540 LP, and 585/65-640 LP) were used. Confocal z-stacks of 10 μm thickness (z-plane distance 1 μm) large-scale images of complete hemispheres were acquired with a 10 × objective. From each animal two slides of a total hemisphere were assessed. Data were acquired and analyzed by investigators blinded to treatment.

For analysis of triple-positive cells (Olig2^+^/APC-CC1^+^/DAPI^+^) nine regions of interest (ROIs) were selected: three specific ROIs in the white matter, three in the cortex and three in the thalamus (each 500 × 500 μm). The 3D-images were converted to maximal intensity projections and converted to Tiff-images followed by analysis with the cell counter plugin of ImageJ (National Institutes of Health, Java 1.8.0). Data are expressed as average positive cells per mm^2^ in the white matter, the cortex and the thalamus.

For analysis of MBP, the positively stained area was analyzed with the binary tool of the NIS AIR software using the NIS Elements AR software 4.0 (Nikon, Germany). Data are presented as percentage of MBP-positive area per hemisphere.

### Behavioral Studies

From P22 on, animals were habituated to the blinded investigator and the inverse 12-h light-dark cycle. Behavioral testing was performed in adolescent animals from P35 to P49 and repeated in adult animals from P137 to P145 (32). Paradigms were chosen as they proved high reliability and validity in our recent studies (10, 25).

One day of open field was followed by 4 days of novel object recognition and further 4 days of Barnes maze test. The open field test (33) assesses anxiety-related behavior and spontaneous motor-activity. For this test, animals were placed into the center of an open field arena (50 × 50 × 40 cm for adolescent or 75 × 75 × 40 cm for adult animals) placed upon an infrared lightbox (emitted light 850 nm, TSE Systems, Bad Homburg, Germany). General motor activity (traveled distance and velocity) was assessed for 5 min. For the novel object recognition test, animals were placed into the center of a Y-maze (arm length: 60 cm; width: 26 cm; wall height: 56 cm) under red light. No external cues were visible from inside the maze. The first day, animals were habituated to the empty arena. On day two and three, they were familiarized with three identical objects (cones), placed at the end of the maze arms. On day four, a familiar object was replaced by a novel one (cylinder). The time spent with the familiar and novel object was recorded. Each session lasted 5 min while only the first 2 min were used for evaluation (34). Spatiotemporal memory was assessed by the Barnes maze (35) as described previously (10). Briefly, animals were placed into the center of the maze (1.22 m width, 0.8 m height, 20 holes at the border, TSE Systems) under red light followed by bright light to allow the animal to recognize extra-maze cues. Animals were expected to explore the maze and find the escape box within 120 s, where they were kept for 1 min. Animals who did not find the escape box were gently placed into it for 1 min. To avoid intra-maze cues due to odor the escape box was rotated clockwise for every other animal, with the same escape location for each animal as on the three training days. On the fourth day of the experiment, all holes were closed and the latency to find the trained escape box was measured (36). All mazes were cleaned with 70% ethanol between trials to eliminate possible odor cues of previous animals. Data were recorded using an automatic tracking system, Ethovision 14 (Noldus, Wageningen, Netherlands).

### Diffusion Tensor Imaging

At the age of 5 months (P151), behavior tested animals were sacrificed and fixed brains were scanned with diffusion tensor imaging. *Ex-vivo* MRI experiments were performed on an actively shielded 9.4T/31cm magnet (Agilent) equipped with 12-cm gradient coils (400 mT/m, 120 μs) with a 2.5 cm diameter birdcage coil. Diffusion weighted images were acquired using a spin-echo sequence with the following parameters: FOV = 23 × 18 mm^2^, matrix size = 128 × 92, 15 slices of 0.6 mm thickness in the axial plane, 3 averages with TE/TR = 45/2000 ms. A total of 36 diffusion weighted images were acquired, 15 of them as b_0_ reference images. The remaining 21 directions were uniformly distributed and non-collinear with a *b*-value = 1,750 s/mm^2^. The diffusion tensor (DT) was computed using DTI-TK (37). The regions of interest (ROI) were manually delineated DTI derived color maps.

ROIs were selected in two different structures of the brain [corpus callosum (CC) and external capsule (EC)] in six different image-planes of the brain from the genu to the splenium of the corpus callosum.

### Statistical Analysis

Statistical analysis was performed with Prism 6 (GraphPad Software, San Diego, CA, USA). Graphical data are presented as median values with boxplots including the 25% and the 75% percentile. Differences between groups were determined by one-way analysis of variance (one-way ANOVA) followed by Bonferroni *post hoc* test for multiple comparison. For DTI results, non-parametric Mann–Whitney *U-*test were used. *p* ≤ 0.05 were considered as statistically significant.

## Results

### A Repetitive Application of Epo Ameliorates Subacute Hyperoxia-Induced Hypomyelination

**Figure 1:**
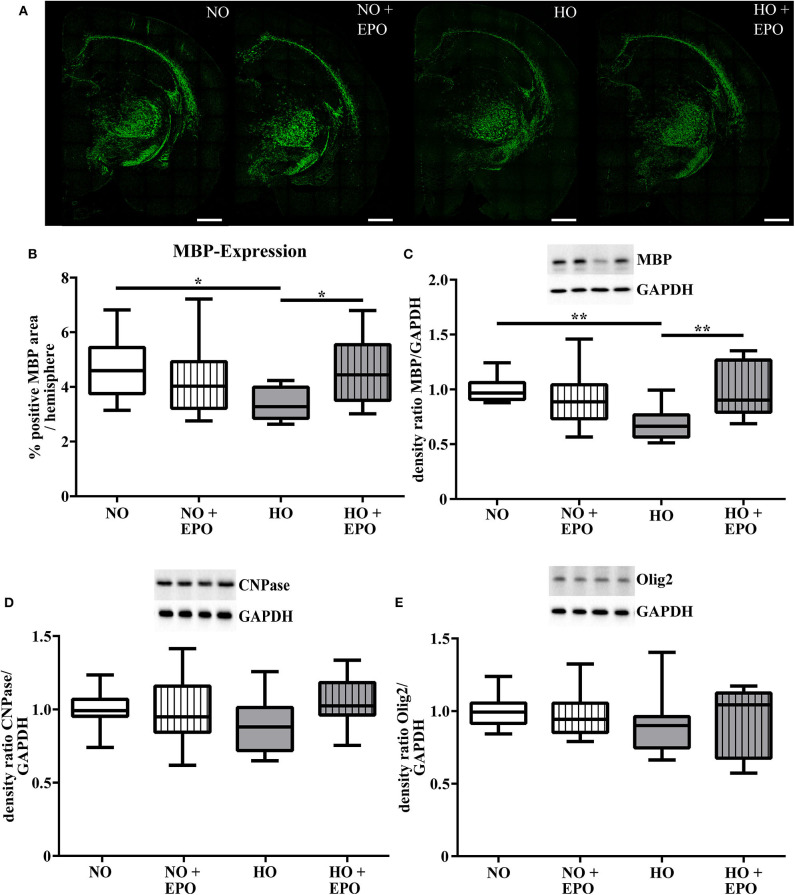
A repetitive application of Erythropoietin (Epo) ameliorates hyperoxia-mediated hypomyelination. Animals were exposed to either normoxia (NO, 21% oxygen) or hyperoxia (HO, 80% oxygen for 48 h from P3 to P5) and were either treated with Epo (4 × 5000 IU/kg body weight per day from P3 to P6) or with an equal amount of normal saline. Analysis of myelination and oligodendrocyte-associated proteins were assessed at P11. **(A)** Representative immunhistochemical images of MBP (green) in coronal brain sections (−3.72 ± 0.7 mm from bregma) on P11. Scale bar = 100 μm **(B)** Analysis of MBP-immunohistochemistry. Data are presented as percentage of MBP-positive area per hemisphere. *n* = 10–11 **(C)** Western blot analysis of MBP-, **(D)** CNPase- and **(E)** Olig2-expression in protein lysates of brain hemispheres (excluding cerebellum). *p < 0.05, **p < 0.01, *n* = 11–12 rats/group.

**Figure 2:**
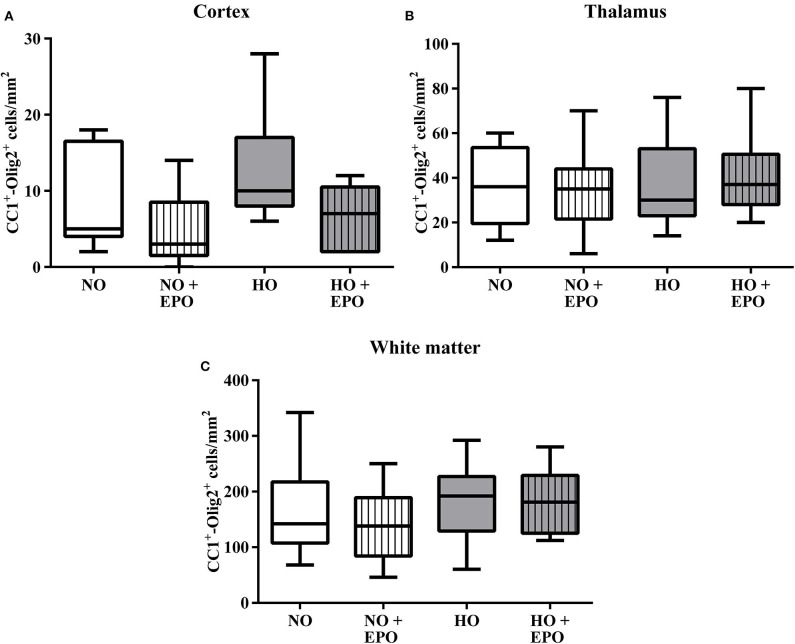
Mature oligodendrocytes were not modulated by hyperoxia and Epo-treatment. The amount of mature oligodendrocytes was analysed via immunohistochemistry for Olig2 and APC-CC1. Triple-positive cells (Olig2^+^/APC-CC1^+^/DAPI^+^) were determined in brain sections from 11-day old rats after exposure to either normoxia [21% oxygen (NO)] or hyperoxia [48 h 80% oxygen (HO)] from P3-P5. Rats were either treated with Epo (4 × 5000 IU/kg body weight per day from P3 to P6) or with an equal amount of normal saline. The average amount of triple-positive cells in **(A)** the cortex, **(B)** the thalamus, and **(C)** the white matter are expressed as cells per mm^2^. *n* = 10–11 rats/group.

MRI studies revealed white matter changes in preterm born infants later in life (4). From our previous experimental studies we know that hyperoxia is one factor causing hypomyelination in the developing brain (8–10, 25). Recently we have shown that a single Epo dose of 20,000 IU/kg body weight led to an improved oligodendrocyte survival acutely after hyperoxia but failed to prevent hypomyelination. According to the clinical setting (23, 24), where repetitive application regimens are common, we adopted our rat model of hyperoxia-induced brain injury and applied 4 × 5,000 IU/kg body weight from P3 to P6. The first dose was administered at the onset of hyperoxia (80% oxygen, 48 h). To analyse the influence of repetitive Epo applications on the developing white matter at P11, we performed immunohistochemical staining for MBP (Figure 1A), APC-CC1, a marker for mature oligodendrocytes, and the pan oligodendrocyte marker Olig2. We found a significant decrease in MBP-expression after hyperoxia, which was attenuated following repetitive Epo treatment (Figure 1B). Analysis of Olig2 and APC-CC1 showed no difference between the 4 study groups (Figure 2). These findings were confirmed by protein analysis via Western blot: While hyperoxia led to a significant decrease of MBP-expression, repetitive Epo-applications reversed this effect in the hyperoxia group (HO + Epo) (Figure 1C). No differences were observed for oligodendrocyte-associated markers Olig2 and CNPase (Figures 1D,E).

### Epo-Treatment Attenuates Neonatal Hyperoxia-Induced Cognitive Deficits in Adolescent and Adult Rats Without Obvious Long-Term Microstructural Alterations in the White Matter

**Figure 3:**
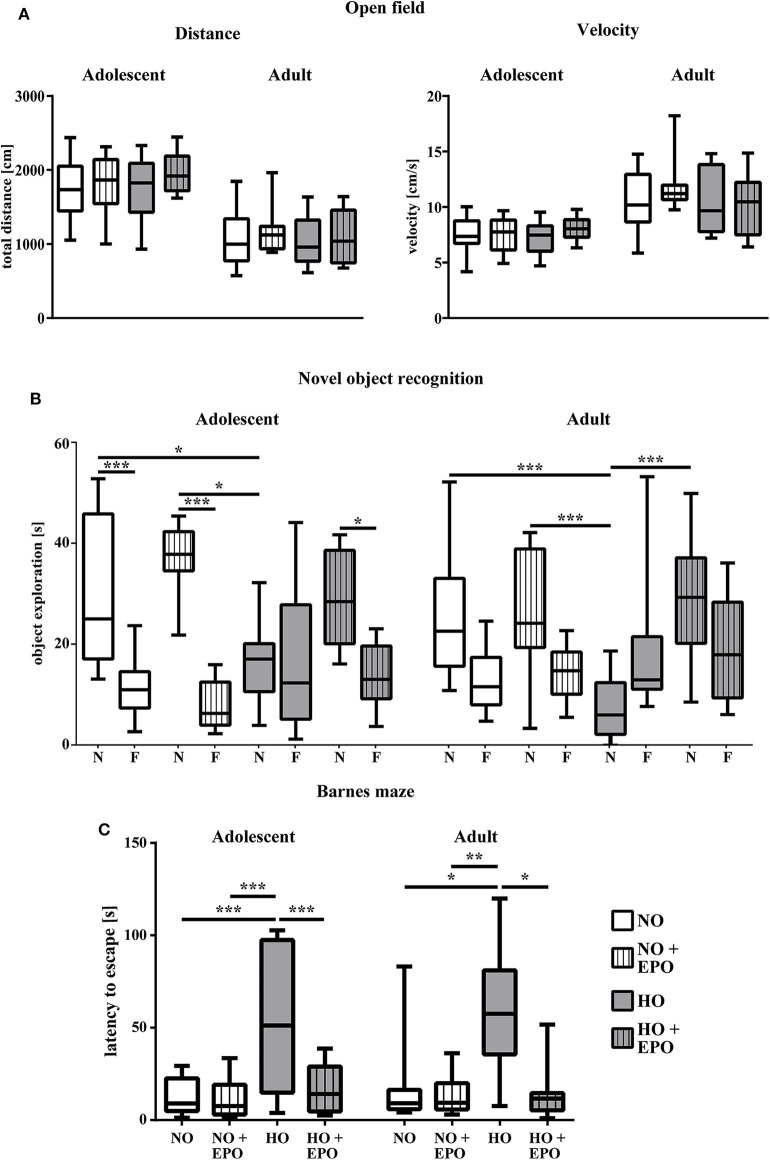
Repetitive Epo treatment improved cognitive function in adolescent and adult rats after neonatal hyperoxia-induced brain-injury. Animals were exposed to either normoxia (NO, 21% oxygen) or hyperoxia (HO, 80% oxygen for 48 h from P3 to P5) and were either treated with Epo (4 × 5000 IU/kg body weight per day from P3 to P6) or with an equal amount of normal saline. Neurodevelopmental outcome was assessed at the age of 2 months (adolescent) and 4 months (adult) **(A)** General motor activity and anxiety-related behavior was tested in the open field, where animals were placed in the maze for 5 min. Movement was assessed by automated video tracking. Motor activity is expressed by the mean velocity and the total distance of the animals. **(B)** Cognitive function was analyzed in the novel object recognition task presented as the exploration time (s) at the novel object (N) vs. familiar objects (F). **(C)** Memory function was determined in the Barnes maze test expressed as the latency to find the trained escape hole after a 3-day training period. *p < 0.05, **p < 0.01, and ***p < 0.001, *n* = 10-12 rats/group.

We showed that repetitive Epo-applications of 4 × 5000 IU/kg body weight in neonatal rats (P3–P6) led to a significant improvement of hyperoxia-induced hypomyelination. To evaluate whether improved myelination on P11 was associated with improved long-term motor-cognitive function we performed a battery of motor-cognitive behavior tests. Anxiety-related behavior and general motor activity were analyzed in the open field test and cognitive function was assessed in the novel object recognition and the Barnes maze test. Figure 3A shows the results of the open field test with the parameters “traveled distance” and “velocity” as indicators of anxiety-related behavior and general motor-activity in adolescent and adult rats. No significant differences were found between study groups. The novel object recognition test is based on the observation, that rats show a preference to novel objects (34). As depicted in Figure 3B, adolescent control animals (NO and NO + Epo) spent significantly more time with the novel object, whereas animals in the hyperoxia group (HO) did not show a clear preference to any object. However, upon Epo-treatment we detected normalized preference for the new, unfamiliar object in adolescent rats. Adult rats exposed to hyperoxia (HO) spent significantly less time with the novel object than the control group (NO and NO + Epo) and the Epo-treated hyperoxic group (HO + Epo). In the Barnes maze, adolescent and adult animals exposed to hyperoxia needed significantly more time to find the trained escape box, while Epo-treatment significantly improved latency to find the box (Figure 3C).

**Figure 4:**
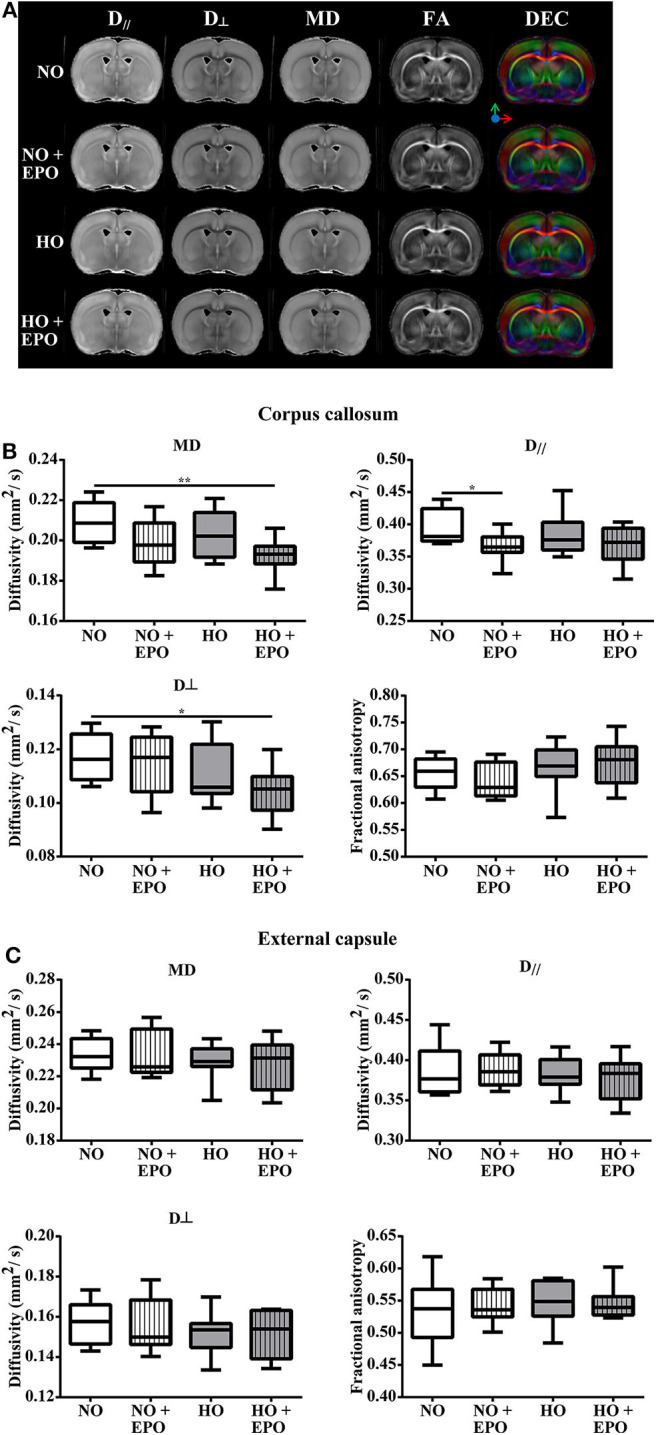
Long-term microstructural development after neonatal hyperoxia-induced brain-injury. Diffusion tensor imaging (DTI) was performed in 5 months old rats, which were exposed to behavior tests at the age of 2 and 4 months. From P3 to P5, rats were either exposed to normoxia [21% oxygen (NO)] or hyperoxia (80% oxygen (HO)] and treated with Epo (4 × 5000 IU/kg body weight per day from P3 to P6) or with an equal amount of normal saline. **(A)** For each treatment group, representative maps for axial diffusivity (AD, *D*_//_), radial diffusivity (RD, *D*_⊥_), mean diffusivity (MD), fractional anisotropy (FA) and direction encoded colour maps (DEC) derived from DTI are shown. Diffusivity values for MD, AD (*D*_//_), RD (*D*_⊥_) and FA are displayed for **(B)** corpus callosum, and **(C)** external capsule. *p < 0.05, **p < 0.01, *n* = 8 rats/group.

To investigate whether improved cognitive outcome might be associated with microstructural alterations of the white matter, post-mortem diffusion tensor imaging was performed in brains of animals that underwent behavioral testing. Analyzing the corpus callosum (CC), we found decreased mean diffusivity (MD) and radial diffusivity (RD, D_D⊥_) in the hyperoxic Epo-treated group (HO + Epo) as well as decreased axial diffusivity (AD, D_//_) in the normoxic Epo-treated group (NO + Epo) compared to controls (NO). Nevertheless, no significant differences for fractional anisotropy (FA) as a marker for white matter integrity (38) were found between study groups. Likewise, no differences for MD, AD, RD, or FA were detected in the external capsule (Figure 4).

## Discussion

The present study investigated the effect of a repetitive Epo dosage regimen on myelination and neurocognitive outcome in a rodent model of hyperoxia-induced brain-injury. With the first application given at the onset of hyperoxia (oxygen 80%, 48 h), a repetitive Epo-treatment with 5,000 IU/kg body weight per day (P3 to P6) improved hyperoxia-induced hypomyelination at term-equivalent age (P11) and long-term neurocognitive outcome in adolescent and adult rats.

The positive effects of Epo on neurocognitive outcome and white matter development have already been described in other models of perinatal brain injury such as hypoxia-ischemia (17, 28, 39, 40). In the preterm model of hyperoxia-induced brain injury, we recently showed that a single Epo application of 20,000 IU/kg bodyweight at the onset of hyperoxia improved neurocognitive outcome in adolescent and adult rats. Nevertheless, in this previous study Epo did not modulate hyperoxia-induced hypomyelination or long-term white matter integrity (25). In the present study, a repetitive Epo application not only improved neurocognitive outcome, but also increased myelination at P11. Clinical studies in preterm infants also showed improved white matter integrity in MRI studies at term-equivalent age (21, 41). It has already been described that Epo promotes maturation and differentiation of oligodendrocytes (19, 29, 42). A direct interaction between oligodendrocytes and Epo may be possible, as oligodendrocytes of all maturation levels have been described to express Epo-receptors (43).

The different effects between a single and repetitive Epo application on myelination on P11 highlight the importance of dosing and timing of Epo-treatment, which varies both in experimental and clinical studies. In the present work, the dosage regimen of 4 × 5,000 IU/kg body weight was based on our previous studies, where the same total amount (20,000 IU/kg body weight) was administered as single bolus application (25, 27). Furthermore, a single dose of 5,000 IU/kg corresponds to a dose of 1,000 IU/kg body weight in preterm infants (44), which is in the range that is commonly used in clinical trials (500–3,000 IU/kg). Improved outcome observed in the present work are supported by a previous study in a model of hypoxic-ischemic brain injury demonstrating that a repetitive dose of 5,000 IU/kg body weight was superior to high-dose Epo treatment regimens in neonatal rats (45). In clinical studies, different treatment regimens have been used, which might explain inconsistent results with regard to Epo's neuroprotective effects in preterm infants. Compared to the placebo group, less moderate to severe disabilities were found by Song et al. in preterm infants <32 weeks of gestation, who received 500 IU/kg body weight at 72 h after birth followed by daily injections for 2 weeks (46). Another important randomized control trial studied the effect of 3 × 3,000 IU/kg Epo, administered within 3 h after birth, at 12 to 18 h and at 36 to 42 h after birth in preterm infants between 26+0 and 31+6 weeks of gestation. Despite promising findings on MRI at term-equivalent age (reduced risk of brain injury and increased structural brain connectivity) in a sub-cohort of this study (21, 47), the Swiss Epo Neuroprotection Trial did not find improved outcome at the age of 2 and 5 years (23, 48). Both studies were part of a recent meta-analysis of four randomized, controlled trials with 1,133 very preterm infants revealing a decreased risk of Mental Developmental Index <70 at the age 18 to 24 months (49). A combined regimen of high dose (6 × 1,000 IU/kg every 48 h) and a chronic application (400 IU/kg until 32 completed weeks of gestation) was used by Juul et al. in a large randomized placebo-controlled trial including only extremely preterm infants (<28 weeks of gestation). Neurodevelopment assessed at the age of 2 years revealed no beneficial effects of Epo treatment (24). However, further follow up at school age and beyond is necessary, to finally evaluate the role of Epo as neuroprotective agent. Neurodevelopmental testing with the Bayley Scales of Infant Development at this early age can predict motor outcome, however it is poorly predictive of later neurocognitive outcome (50). This is in line with the retrospective study by Neubauer et al. indicating improved neurodevelopmental outcome in extremely low birth infants at school age (20). Our pre-clinical results confirm these long-term effects with improved cognitive outcome even at adulthood. Taking these concerns into account, the Swiss study group has already planned a prospective follow-up study aiming to test executive function in their cohort at the age of 7–12 (48, 51). In addition to dosage, timing of Epo applications might be a further explanation for inconsistent study results. This seems to be supported by our findings demonstrating improved myelination at term-equivalent age after repetitive treatment but not after a single bolus administration of the same cumulative dose (25). However, it needs to be considered that compared to our previous study, we also changed the timing of hyperoxia with an earlier onset at P3. We intended to adopt our model to the group of extremely preterm infants (<28 weeks of gestation), as they are most vulnerable to white matter injury. In rats, this period is around P3 and characterized by the abundance of oligodendrocyte progenitors. Around P6 immature oligodendrocytes, which are less vulnerable, are predominant (52). These differences in maturation possibly explain the different effect on myelination between this study and our previous work (25).

Several studies have described that hyperoxia induces acute cell death with a reduction of immature and mature oligodendrocytes in the developing brain (7, 9, 53). Nevertheless, this reduction–as well as the reduction of MBP–is likely to be transient. In mice exposed to 48 h hyperoxia (80% oxygen) from P6 to P8, Schmitz et al. have described recovery of total and mature oligodendrocyte populations 4 days after insult while MBP-values were still reduced (54). This might explain our findings with reduced MBP-values on P11, which were not accompanied by a decreased number of mature oligodendrocytes and associated proteins. Neonatal hyperoxia induces long-term microstructural alterations of the white matter as previously shown in pre-clinical DTI studies. These studies revealed a reduced fractional anisotropy in different white matter structures (e.g., corpus callosum, external capsule) indicating disturbed white matter integrity (9, 25, 54). Interestingly, we could not replicate these findings in the present study even though animals exposed to neonatal hyperoxia showed long-term impaired cognitive function. The fact that the animals in this study were older (P151) compared to previous studies might have contributed to these results. Nevertheless, normal white matter integrity on DTI does not exclude abnormalities on the cellular level. Ultrastructural changes assessed via electron microscopy including disturbed long-term axon-oligodendrocyte integrity after neonatal hyperoxia-induced brain injury have been described (55, 56).

Limitations of our study include that we did not investigate long-term abnormalities of the white matter beyond the microstructural level. Future studies should more specifically analyse alterations at the cellular level. Though we detected changes regarding cellular degeneration and survival factors in previous studies (16, 25–27, 57), detailed molecular analyses should be addressed to better understand the mechanisms underlying the neuroprotective effect of Epo in this experimental setting. Furthermore, due to study design no detailed immunohistochemical analysis of oligodendrocyte degeneration or different oligodendrocyte populations was performed immediately after hyperoxia. Thus, we can only speculate that our findings (similar numbers of mature oligodendrocytes in both control and hyperoxia group) are the result of recovery processes (54). Recovery processes might also explain our DTI results as studies investigating earlier time points found an altered microstructure (9, 10, 25). Although in this study we focused on long-term outcome, serial MRI studies could provide this information in subsequent studies. A further limitation is that we only investigated cerebral structures, despite evidence from animal studies that hyperoxia has detrimental effects on the developing cerebellum (58, 59). As impaired neurocognitive outcome of preterm infants is associated with cerebellar injury (60), the effects of Epo on the cerebellum need to be elucidated in the future.

Nevertheless, the findings of this study are of importance, given that the exposure of extremely preterm infants to hyperoxia is almost unavoidable during their treatment in the neonatal intensive care unit. Hyperoxia increases their risk of long-term neurodevelopmental impairment in later life. Thus, understanding of the pathophysiology of hyperoxia-induced brain injury is required as well as the investigation of potential preventive or treatment options such as Epo. Despite disappointing results in first clinical trials (23, 24) our results underline the importance of long-term follow-up studies for neurocognitive outcome in these cohorts.

To conclude, the objective of this study was to investigate the effect of repetitive Epo treatment on cerebral white matter and motor-cognitive outcome in a rodent model of hyperoxia-induced brain injury. We found that a dosage regimen close to clinical application improved myelination and long-term neurocognitive outcome. Despite inconsistent results from recent clinical studies, Epo remains a promising neuroprotective agent in the prevention and therapy of preterm brain injury. The results of the long-term follow-up of recent clinical trials have to be awaited.

## Data Availability Statement

The raw data supporting the conclusions of this article will be made available by the authors, without undue reservation.

## Ethics Statement

The animal study was reviewed and approved by State Agency for Nature, Environment and Consumer Protection (LANUV), North Rhine-Westphalia.

## Author Contributions

MD, MS, JF, UF-M, and IB: conceptualization. MD, MS, SE, JH, and IB: data curation. MD, MS, YL, and IB: formal analysis. MD, UF-M, IB, and YL: funding acquisition. MD, MS, MK, MH, YL, and IB: investigation. MD, MS, MK, MH, YL, and IB: methodology. MD, MS, JF, SS, JH, and IB: validation. All authors contributed to manuscript preparation, read, and approved the submitted version.

## Conflict of Interest

The authors declare that the research was conducted in the absence of any commercial or financial relationships that could be construed as a potential conflict of interest.
